# TFII-I-mediated polymerase pausing antagonizes GLI2 induction by TGFβ

**DOI:** 10.1093/nar/gkaa476

**Published:** 2020-06-16

**Authors:** Angela L McCleary-Wheeler, Brooke D Paradise, Luciana L Almada, Annika J Carlson, David L Marks, Anne Vrabel, Renzo E Vera, Ashley N Sigafoos, Rachel L Olson, Martin E Fernandez-Zapico

**Affiliations:** Schulze Center for Novel Therapeutics, Mayo Clinic, Rochester, MN 55905, USA; Mayo Clinic Graduate School of Biomedical Sciences, Mayo Clinic, Rochester, MN 55905, USA; Schulze Center for Novel Therapeutics, Mayo Clinic, Rochester, MN 55905, USA; Mayo Clinic Graduate School of Biomedical Sciences, Mayo Clinic, Rochester, MN 55905, USA; Schulze Center for Novel Therapeutics, Mayo Clinic, Rochester, MN 55905, USA; Schulze Center for Novel Therapeutics, Mayo Clinic, Rochester, MN 55905, USA; Schulze Center for Novel Therapeutics, Mayo Clinic, Rochester, MN 55905, USA; Schulze Center for Novel Therapeutics, Mayo Clinic, Rochester, MN 55905, USA; Schulze Center for Novel Therapeutics, Mayo Clinic, Rochester, MN 55905, USA; Schulze Center for Novel Therapeutics, Mayo Clinic, Rochester, MN 55905, USA; Schulze Center for Novel Therapeutics, Mayo Clinic, Rochester, MN 55905, USA; Schulze Center for Novel Therapeutics, Mayo Clinic, Rochester, MN 55905, USA

## Abstract

The modulation of GLI2, an oncogenic transcription factor commonly upregulated in cancer, is in many cases not due to genetic defects, suggesting dysregulation through alternative mechanisms. The identity of these molecular events remains for the most part unknown. Here, we identified TFII-I as a novel repressor of GLI2 expression. Mapping experiments suggest that the INR region of the GLI2 promoter is necessary for GLI2 repression. ChIP studies showed that TFII-I binds to this INR. TFII-I knockdown decreased the binding of NELF-A, a component of the promoter–proximal pausing complex at this site, and enriched phosphorylated RNAPII serine 2 in the GLI2 gene body. Immunoprecipitation studies demonstrate TFII-I interaction with SPT5, another pausing complex component. TFII-I overexpression antagonized GLI2 induction by TGFβ, a known activator of GLI2 in cancer cells. TGFβ reduced endogenous TFII-I binding to the INR and increased RNAPII SerP2 in the gene body. We demonstrate that this regulatory mechanism is not exclusive of GLI2. TGFβ-induced genes CCR7, TGFβ1 and EGR3 showed similar decreased TFII-I and NELF-A INR binding and increased RNAPII SerP2 in the gene body post-TGFβ treatment. Together these results identify TFII-I as a novel repressor of a subset of TGFβ-responsive genes through the regulation of RNAPII pausing.

## INTRODUCTION

GLI2 is a zinc-finger transcription factor belonging to the GLI family of proteins. Highly regulated processes make it a vital protein for normal development, and loss of GLI2 results in late embryonic or immediate prenatal death (1,2). However, GLI2 has also been well documented as an important oncogene, and its overexpression has been demonstrated in a variety of tumors (3–11). *In vivo* models have shown that GLI2 overexpression alone can drive cancer development (4,10). Interestingly, increased expression levels of GLI2 are rarely explained by GLI2 gene mutations, and few reports have documented the amplification of GLI2 in tumors (12,13). Thus, other mechanisms must exist to account for increased GLI2 gene expression in cancer cells.

In our studies, we evaluated the function of TFII-I, an INR-binding transcription factor encoded by the GTF2i gene, on GLI2 gene transcription (14–16). TFII-I is a ubiquitously expressed transcription factor that has the ability to either repress or activate transcription of target genes in a context-dependent and isoform-dependent manner (14,17–19). The activity of TFII-I is signal-induced, and the mechanisms of this induction have been well studied (20). However, what occurs following TFII-I binding to target genes and specifically how it modulates the expression of these target genes following transcriptional initiation is not well understood. Studies have demonstrated that TFII-I can interact with HDACs and members of the PRC complex to modulate gene repression in specific cellular contexts, but little else is understood in regard to TFII-I regulation of chromatin dynamics (18,21–25). We have found that TFII-I binds to the INR of the GLI2 promoter under endogenous conditions and functions as a repressor of GLI2 transcriptional activation. The mechanism of repression mediated by TFII-I was found to be mediated by RNA polymerase II (RNAPII) pausing, as levels of phosphorylated RNAPII serine 2 (RNAPII SerP2) increased in the GLI2 gene concurrent with decreases in RNAPII pausing complex binding in the promoter following TFII-I knockdown. In addition, TFII-I is able to antagonize TGFβ induction of GLI2 transcription. Further studies demonstrated a decrease in RNAPII pausing complex components and TFII-I at the GLI2 promoter after treatment with TGFβ, and a simultaneous increase in RNAPII SerP2 in the GLI2 gene body. Finally, RNA-sequencing studies identified an additional set of TGFβ-induced genes which experience the same mechanism of regulation. Thus, we report a novel mechanism of GLI2 transcriptional repression through TFII-I and show for the first time that TFII-I acts as a modulator of polymerase pausing. Further, we have shown this mechanism of gene regulation may be applicable to a larger cohort of TGFβ-responsive genes.

## MATERIALS AND METHODS

### Cell culture conditions, plasmids and reagents

PANC1 and HepG2 cell lines were obtained from ATCC. These cells lines were chosen both for disease relevance and the high (PANC1) or low (HepG2) endogenous expression of GLI2. PANC1 cells were grown in DMEM medium with 10% fetal bovine serum (FBS), and HepG2 cells in MEM with 10% FBS. Plasmids utilized for experiments included a 3xFLAG-TFII-I expression vector corresponding to the TFII-I isoforms α, β, δ and γ (GenScript, Piscataway, NJ) and SPT5-HA in the p3xFLAG-CMV14 vector (original SPT5 sequence from Capital Biosciences in pORF). The 8xGLI-luciferase reporter was a gift from Dr Chi-chung Hui (University of Toronto, Toronto, Ontario, Canada). The GLI2 promoter constructs were kindly provided by Dr Alain Mauviel (Institut Curie, INSERM U1021/CNRS UMR334, Paris, France). Preparations and descriptions of the GLI2 promoter reporter constructs −1624, −454, −119 and −29 have been previously reported (26). A mutant −29 GLI2 reporter was generated using the QuickChange site-directed mutagenesis kit (Agilent Technologies, Santa Clara, CA, USA). In this, the GLI2 INR (TCATTCT) was changed to a TATA box (TTATAAT) using the following primers for mutagenesis: 5′-GGTGTCTGGGATTTCAGGTTTCAGGGTGATTCGCTTATAATGCTCTGATTACTAATTTAT-3′ (sense); 5′-ATAAATTAGTAATCAGAGCATTATAAGCGAATCACCCTGAAACCTGAAATCCCAGACACC-3′ (antisense). *In silico* sequence analysis of the promoter reporter constructs revealed no cryptic transcriptional start sites or the presence of mutations to the promoter region or parental contruct backbone (Promega, Madison, WI). Recombinant human TGFβ1 ligand (rhTGFβ1; R&D Systems, Minneapolis, MN) was reconstituted in sterile 4 mM HCl with 1 mg/ml bovine serum albumin to a final concentration of 2 ng/μl.

### rhTGFβ1 treatments

HepG2 cells were seeded the day prior to treatment as indicated for RNA expression evaluation and ChIP assays. Growth medium was removed from the cells and plates were washed twice with sterile PBS. MEM medium with no serum was added to the cells. A total of 5 ng of rhTGFβ1 per ml of medium was added to the treatment plates, or an equal volume of the vehicle only was added to control plates.

### Transfection

For TFII-I overexpression studies, PANC1 and HepG2 cells were transfected using electroporation (BTX Harvard Apparatus, Holliston, MA) with 1 pulse of 260 V for 25 mS. For TFII-I overexpression experiments, 9 μg of expression construct was used per 3 × 10^6^ cells in 400 μl of OPTI-MEM serum-free medium (Invitrogen, Grand Island, NY). The electroporated cells were then plated on 10 cm^2^ plates with 10 ml of growth medium and cells were harvested for expression assays 24 hours later. HepG2 cells used in SPT5-HA overexpression experiments were transfected using X-tremeGene reagent (Roche Applied Science, Penzburg, Germany). Briefly, 0.5–1.0 × 10^6^ cells were plated on 10 cm^2^ plates 24 h prior to transfection. 3 μg of DNA and 12 μl of X-tremeGene HP reagent were diluted in 400 μl of OPTI-MEM reagent for each plate to be transfected and added to cells according to the manufacturer ’s protocol. Cells were then harvested 48 hours later for immunoprecipitation studies. Empty vector was used as a control.

TFII-I knockdown was performed using siRNA specific for TFII-I, Hs_GTF2I_15 FlexiTube (siRNA 2) and Hs_GTF2i_18 FlexiTube (siRNA 1) (Qiagen, Germantown, MD) along with a non-targeting siRNA negative control (Qiagen Allstars Negative Control siRNA). siRNA 2 was used throughout the entirety of this manuscript, and siRNA 1 was used for validation purposes. 20 nM of targeting or non-targeting (NT) siRNA was transfected into HepG2 cells using Oligofectamine (Invitrogen) or RNAiMax (Thermo Fisher Scientific, Waltham, MA). 0.5 × 10^6^ HepG2 cells were plated on 10 cm^2^ culture plates 24 h prior to transfection. 20 nM siRNA or NT siRNA was mixed with 400 μl of OPTI-MEM in one tube while 12 μl of Oligofectamine was diluted in 400 μl of OPTI-MEM in a separate tube. After 5 min, the two tubes were combined, mixed gently, and allowed to incubate for another 30 min. The complexes were then added to plates of cells containing 4 ml of serum-free MEM and incubate at 37°C. After 4 h, 2 ml of MEM with 30% FBS was added to the plates and the cells were incubated at 37°C for 48 h prior to harvesting.

### Luciferase reporter assay

Cells were grown and transfected as described above. Transfected cells were plated in triplicate in six-well plates. Lysates were obtained and analyzed for luciferase signal as per the manufacturer's protocol (Promega, Madison, WI). Cells were harvested 24 h post-transfection for overexpression studies while cells transfected for knockdown studies were harvested 48 h post-transfection. Empty vector or NT siRNA were used as controls, respectively. To account for variation among samples, the total protein in each well was quantified using the BioRad protein assay (BioRad, Hercules, CA, USA). The luciferase results were then normalized to the determined total protein levels.

### Quantitative reverse transcription PCR (RT-qPCR)

Total RNA was extracted from cells using TRIzol reagent (Invitrogen, Grand Island, NY, USA). RNA was reverse transcribed to cDNA using the High Capacity cDNA kit (Applied Biosystems, Foster City, CA, USA). The resultant cDNA was amplified by PCR using a quantitative method. The following sense and antisense primer sets were used:

TFII-I: 5′-GTGGCCCCATCAAAGTGAAAACTG-3′(sense); 5′-CGAAGTTGAACTCCCTCACTTTCC-3′ (antisense); CCR7: 5′-GGGACCTGAGGGTCAGGATA-3′ (sense); 5′-CTTGACACAGGCATACCTGGAA-3′ (antisense); TGFβ1: 5′-AGCAACAATTCCTGGCGATACCTC-3′ (sense); 5′-GAAAGGCCGGTTCATGCCATGAAT-3′ (antisense); SHH: 5′-TCCAGAAACTCCGAGCGATTTAAG-3′ (sense); 5′-TCACTCCTGGCCACTGGTTCA-3′ (antisense); EGR3: 5′-GGTGACCATGAGCAGTTTGC-3′ (sense); 5′-ACCGATGTCCATTACATTCTCTGT-3′ (antisense); GAPDH: 5′-GACCTGACCTGCCGTCTAGAAAAA-3′(sense);
5′-ACCACCCTGTTGCTGTAGCCAAAT-3′ (antisense); HPRT: 5′-CCTGGCGTCGTGATTAGTGAT-3′ (sense); 5′-AGACGTTCAGTCCTGTCCATAA-3′ (antisense); TBP: 5′-TATAATCCCAAGCGGTTTGC-3′ (sense); 5′-CCCAACTTCTGTACAACTCTAGCA-3′ (antisense). Real-time expression studies of GLI2 (Hs_01119974-m1), TFII-I (Hs_01073660_m1), and GAPDH (Hs_02758991_g1) were performed using TaqMan primer/probes and the ABI ViiA-7 quantitative thermocycling unit (Applied Biosystems). 1 μg of RNA was transcribed to cDNA using the High Capacity cDNA synthesis kit, and 2 μl of cDNA from samples was used for the resultant qPCR. The amount of GLI2 or TFII-I transcript was expressed as the relative difference to the control gene (GAPDH) and treatment group using the ΔCt method of relative quantitation.

### Immunoblotting

HepG2 cells were plated at 1 × 10^6^ cells per 10 cm^2^ plate and transfected as described above with siRNA or expression vectors. Forty eight hours following transfection, cells were harvested and lysed with a high salt NP-40 buffer (25 mM HEPES, 400 mM NaCl, 5 mM EDTA, 0.5 mM CaCl2, 0.2% NP-40, 0.2% Tween-20, 10% glycerol) with complete protease inhibitor cocktail (Roche Applied Science Penzberg, Germany). Cells were vortexed following the addition of the buffer and snap frozen at −80°C until analysis. Samples were thawed on ice and nuclei were broken by shearing samples through a 27 $\frac{1}{2}$-gaugue needle 20 times. Samples were then centrifuged at 17 000 × *g* for 30 min and the supernatant transferred to a clean microcentrifuge tube. Quantification of the protein was performed using a BCA-based kit with a BSA standard curve (Thermo Fisher Scientific). 100 μg of protein from each sample was loaded onto 5% polyacrylamide gels and separated by electrophoresis. The proteins were then transferred to PVDF membranes (EMD Millipore, Bedford, MA). Membranes were then blocked with 5% milk in TBST and incubated in primary antibody overnight at 4°C on a rocker platform. Membranes were washed three times for 10 min each in TBST and then incubated with a secondary donkey anti-rabbit IgG or sheep anti-mouse IgG linked to HRP (GE Healthcare, UK) at room temperature with rocking for 1 h. Membranes were again washed as described and the protein bands were visualized with SuperSignal ECL detection kit (Thermo Fisher Scientific) and radiographic film exposure. Primary antibodies used include GLI2 (Cell Signaling, Danvers, MA), TFII-I (Bethyl, Montgomery, TX), and α-Tubulin (Sigma-Aldrich, Saint Louis, MO, USA).

### Chromatin Immunoprecipitation (ChIP) assay

Cells were plated on 15 cm^2^ plates and treated as indicated. The cells were crosslinked with 1% formaldehyde for 10 min at room temperature. Remaining formaldehyde was then quenched with 125 mM glycine at room temperature for 5 min. The medium was then aspirated from the cells, and the cells were washed twice with cold 1X PBS. After the final wash, the plates were placed on ice and 1 ml of cold PBS with complete protease inhibitor cocktail (Roche Applied Science) was added to each plate of cells. The cells were scraped from the plates, transferred to microcentrifuge tubes, and were pelleted by centrifuging at 4°C and 800 × *g* for 5 minutes. The supernatant was carefully aspirated and the remaining cell pellets were flash frozen in liquid nitrogen and then stored at −80°C until use. Cell pellets were thawed on ice, lysed in cold cell lysis buffer, and incubated on ice for 15 min with vortexing every 5 min. The suspensions were then centrifuged at 800 × *g* at 4°C for 5 min. The supernatant was removed and the remaining nuclear pellet was lysed in cold nuclear lysis buffer. The chromatin was then sheared physically using 30 cycles of 30 s on/ 30 s off sonication with a Bioruptor UCD-300 (Diagenode, Denville, NJ). Chromatin was fragmented to lengths between 200 and 1000 bp. Samples were precleared with magnetic protein G beads (Dynabeads, Invitrogen) and normal IgG (mouse or rabbit, Millipore) with rotation at 4°C for 1–2 h. The beads were pelleted with a magnet and the supernatant was transferred to a new microcentrifuge tube. Two percent of the sample was removed for use as an input sample and stored at 4°C. The remaining sample was combined with magnetic protein G beads pre-bound to appropriate antibody and rotated overnight at 4°C to immunoprecipitate the protein of interest along with the crosslinked chromatin. The antibodies used for ChIP included TFII-I (Bethyl), RNAPII CTD (Millipore), RNAPII SerP5 (Abcam), RNAPII SerP2, (Abcam) and NELF-A (Santa Cruz Biotechnology). The following day, the beads were pelleted with a magnet and washed using a series for four buffers (Low salt wash buffer, high salt wash buffer, lithium chloride wash buffer and TE buffer). The samples were eluted by adding 100 μl of elution buffer and 1 μg Proteinase K to the beads and incubating for 2 h at 62°C followed by 10 min at 90°C. The samples were cooled to room temperature, and DNA was purified using column purification kit (IBC, MidSci, Saint Louis, MO). PCR was performed using the following primer sets:

GLI2 INR: 5′-AAGAAACCAGGTGGCGGGAGGGTG-3′ (sense); 5′-GATCACAGATGCGGTGCCTTGAAC-3′ (antisense)
GLI2 INR/SMAD3: 5′-TGTGACTTTAATGCGGTGTGCACG-3′ (sense); 5′-GATCACAGATGCGGTGCCTTGAAC-3′ (antisense)
GLI2 Gene Body Set 1: 5′-AGAGTCTCACTCTGTCTCCAA-3′ (sense); 5′-GTCCCTTCTGGCTTCCAAATA-3′ (antisense)
GLI2 Gene Body Set 2: 5′-AGCCATCCCTGGAGAGAC-3′ (sense); 5′-GAGCCAAGAGGCTGTGTAAAT-3′ (antisense)
GLI2 Gene Body Set 3: 5′-GGCTCTGTGTACTATCTTCTTCTC-3′ (sense); 5′-AAATGCCTCCTGACACCTC-3′ (antisense)
CCR7 INR: 5′-GTGGCTTCTCCGACAACTTA-3′ (sense); 5′-TTCTCACATGAAGAGGCTCAC-3′ (antisense)
CCR7 Gene Body Set 1: 5′-GTTGTGAGAATGGTGCGGTG-3′ (sense); 5′-AGCCAGATCAAAGCAGGTGG-3′ (antisense)
CCR7 Gene Body Set 2: 5′-AGACCAGGCTGAGGCTAAGA-3′ (sense); 5′-ATCAAGGAGGCTGTGGTGTG-3′ (antisense)
CCR7 Gene Body Set 3: 5′-CCCCAGACTAGGTTTAGGGG-3′ (sense); 5′-ACCGTTGGGGCTCTCTCAAG-3′ (antisense)
TGFβ1 INR: 5′-CGACATGGAGCTGGTGAAG-3′ (sense); 5′-CGGGTGCTGTTGTACAGG-3′ (antisense)
TGFβ1 Gene Body Set 1: 5′-TGTCCGAAAGAGGATGGCAC-3′ (sense); antisense: 5′-CGGTCCACTTCGCTATCTCC-3′ (antisense)
TGFβ1 Gene Body Set 2: 5′-AGGGACGGGAGGTTATTGGA-3′ (sense); 5′-GTGAAACACCGAGGACACCT-3′ (antisense)
SHH INR: 5′-AAGAGAGAGCGCACACG-3′ (sense); 5′-CTCCTCTTCCCGAACCC-3′ (antisense)
SHH Gene Body Set 1: 5′-ACGAGAAGCCGAACACTTCC-3′ (sense); 5′-CCACGTCTGTTACCGTCCTC-3′ (antisense)
EGR3 INR: 5′-GCTCTCCTAACGCAAACCT-3′ (sense); 5′-GAAGAGAGAGGAAAGAAGGATACAG-3′ (antisense)
EGR3 Gene Body Set 1: 5′-CGAGAAGGCTAGGTTGGCG-3′ (sense); 5′-AGAGCGCGGGTGAAAAAGAC-3′ (antisense)
EGR3 Gene Body Set 2: 5′-GCTCCGGGTCTGAAACTACC-3′ (sense); 5′-GAAGCATTGTTGTTCTTCCCGA-3′ (antisense)

All primer sets were validated for qPCR and were used at 300 nM concentration with SYBR Green master mix (Quanta Biosciences, Gaithersburg, MD) and run on a Bio-Rad CFX384 unit at the following conditions: 95°C × 5 min; 95°C × 30s, [AT]°C × 45 s, 72°C × 1 min for 40 cycles followed by a melt curve analysis. [AT] represents the annealing temperature, which was optimized for each primer set. Temperatures ranged from 56 to 64°C. The results were quantified and graphed either as percentage of input or fold of enrichment.

### Flavopiridol treatment

HepG2 cells were plated at a density of 150 000 cells per 10 cm^2^ plate 24 h prior to treatment. The cells were treated with 150 μM of flavopiridol in DMSO (Sigma-Aldrich) or an equal amount of DMSO as a control. The cells were harvested 6 h after treatment. RNA was isolated and RT-qPCR was performed for TFII-I, GLI2, and GAPDH as described above.

### Immunoprecipitation

For immunoprecipitation, HepG2 and PANC1 cells transfected with FLAG- and HA-tagged expression constructs were washed twice in PBS, and lysed at 4°C in IP lysis buffer (50 mM Tris, pH 7.4, 1% NP-40, 2.5% glycerol with complete protease inhibitor cocktail (without EDTA) with 400 mM NaCl. Samples were passed 5 times through a 27 $\frac{1}{2}$-gauge needle using a 1 ml syringe and then diluted with IP lysis buffer to 150 mM NaCl. After the lysates were cleared at 15 000 × *g* for 10 min, supernatants were collected and subjected to immunoprecipitation following the Dynabeads Protein G immunoprecipitation kit protocol. The supernatants were incubated with immunopreciptating antibodies for 16 h at 4°C with rotation. Pellets were washed with lysis buffer and immunoprecipitates were eluted with 2× SDS-PAGE sample buffer including dithiothreitol and analyzed by western blotting using rabbit anti-TFII-I (Bethyl), mouse anti-FLAG (Sigma), or anti-HA (Sigma).

### RNA-sequencing analysis

Sequencing and mapping were completed using the services of the Mayo Clinic Genome Analysis Core. Sequencing library was prepared using TruSeq v2. Sequencing platform: HiSeq PE 51 base reads, 300 million reads per lane, and four samples were run per lane. Samples were aligned to hg19 human reference genome. Reads were analyzed using MAPRseq v.2.0.0 against the hg19 mouse reference for count calculation. Differential expression was calculated using edgeR. Genes were filtered for FDR >0.05. Fold change of gene expression was calculated in Excel using gene counts from the treatment group and dividing by the respective vehicle group. Fold change is portrayed as log_2_(FC) for ease of understanding. *P*-values were calculated in excel from biological replicates that were sequenced. Excel was used to create the volcano plot, in which the log of the *P*-value is portrayed as a function of the log_2_(FC). GraphPad Prism was used to create additional graphs representing the RNA sequencing results. GEO Accession Number: GSE139021.

### Statistical analysis

Results were graphed as mean values ± SD of three independent biological replicates. Mean values were normalized to respective controls using Microsoft Excel. All experiments were performed a minimum of three times. The Student's *t*-test was used to assess statistical significance across biological replicates, with an asterisk representing *P* < 0.05. Graphs were created and statistical analysis performed using Prism software.

## RESULTS

### TFII-I represses GLI2 transcriptional activation by directly decreasing GLI2 promoter activity

**Figure 1.**
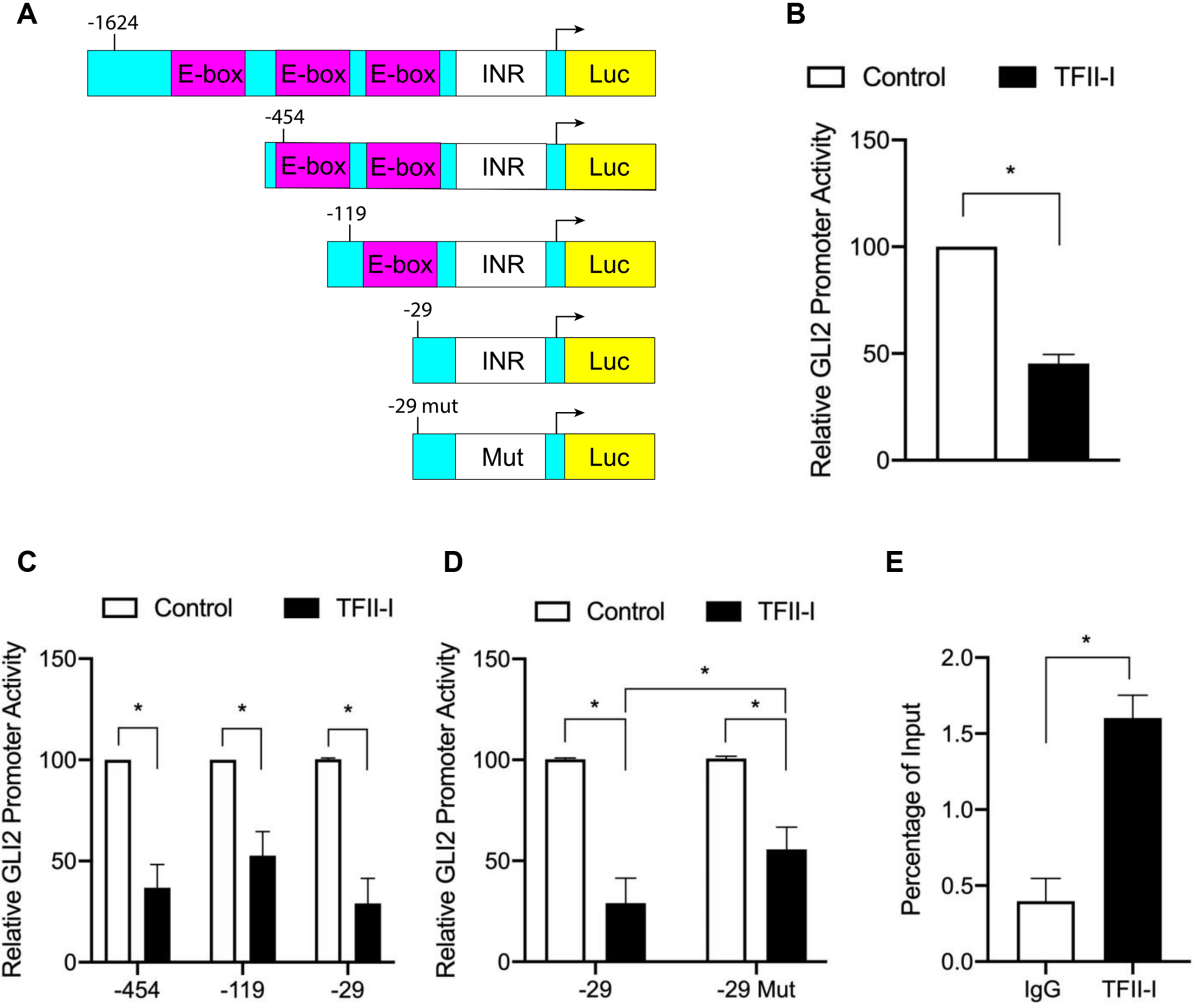
TFII-I represses GLI2 promoter activity through binding to the GLI2 INR. (**A**) Diagram showing the locations of E-boxes (containing multiple E-box sites) and INR in the GLI2 promoter construct and deletion constructs used for promoter activity studies. (**B**) Co-expression of TFII-I and a GLI2 reporter containing ∼1600 bp of the GLI2 promoter in PANC1 cells shows repression of reporter activity. (**C**) Multiple GLI2 reporter constructs were used in PANC1 cells to help localize the promoter region responsible for TFII-I repressive activity. Reporter fragments included an intact INR with or without additional sequence upstream. (**D**) The -29 fragment was compared to a −29 reporter in which the INR was mutated to a TATA box to determine INR necessity for TFII-I activity in PANC1 cells. (**E**) ChIP analysis confirmed binding of TFII-I to the GLI2 INR region under basal conditions in PANC1 cells. For this figure, *N* = 3 and the asterisk represents *P* ≤ 0.05.

Given that little is known about the mechanisms of transcriptional regulation of GLI2, we started with an *in silico* analysis of the GLI2 promoter to evaluate regulatory elements. The GLI2 gene has a TATA-less promoter, and is defined by the presence of an initiator element (INR) (26). We also identified several E-box elements within 2000 bp upstream of the GLI2 transcription start site (Figure 1A, each box shown contains multiple E-box sites). TFII-I is a known E-box and INR-binding transcription factor, so we wanted to test whether TFII-I could influence GLI2 transcription, and which segments of the GLI2 promoter were crucial for regulation by TFII-I. Given the extensive characterization of the TFII-I δ isoform in existing literature (14–20), we used this variant for our experiments and refer as TFII-I for the remaining of the manuscript. The activity of the full promoter (−1624) segment was repressed when TFII-I was overexpressed (Figure 1B). To determine required sequence motifs within this full-length region, we used various truncations of the promoter region. The −454 reporter still contains E-box elements in addition to the INR. Most of the E-box elements are eliminated in the −119 reporter, with only the INR and one E-box region remaining. The smallest reporter, −29, contains only the INR (Figure 1A). Co-expression of TFII-I along with each of these truncated GLI2 reporters revealed repression of promoter activity (Figure 1C). We hypothesized that the INR is the required sequence motif for TFII-I binding to repress the GLI2 promoter. To test this hypothesis, we mutated the GLI2 INR in the −29 reporter construct to a TATA box (−29mut). While TFII-I is able to still repress the activity of the −29mut reporter construct, the repression is partially relieved when compared to the −29 reporter containing the full GLI2 INR (Figure 1D). Next, we used ChIP assay to confirm TFII-I binding to GLI2 promoter at the INR region. Figure 1E shows endogenous binding of TFII-I to this region of the GLI2 gene. To control for the specificity of this interaction, we examined TFII-I binding to the three independent regions in the GLI2 gene body, the data included in Supplemental Figure S1A shows that TFII-I binding to this area is 4-fold lower and closer to the background control. Lastly, we determined if other TFII-I isoforms behave similar to the δ isoform. We co-expressed full length GLI2 promoter luciferase reporter construct and all four TFII-I isoforms (α, β, δ and γ) (27) in PANC1 cells. When compared to the control, the overexpression of each TFII-I isoform can repress GLI2 promoter activity (Supplemental Figure S2B).

**Figure 2.**
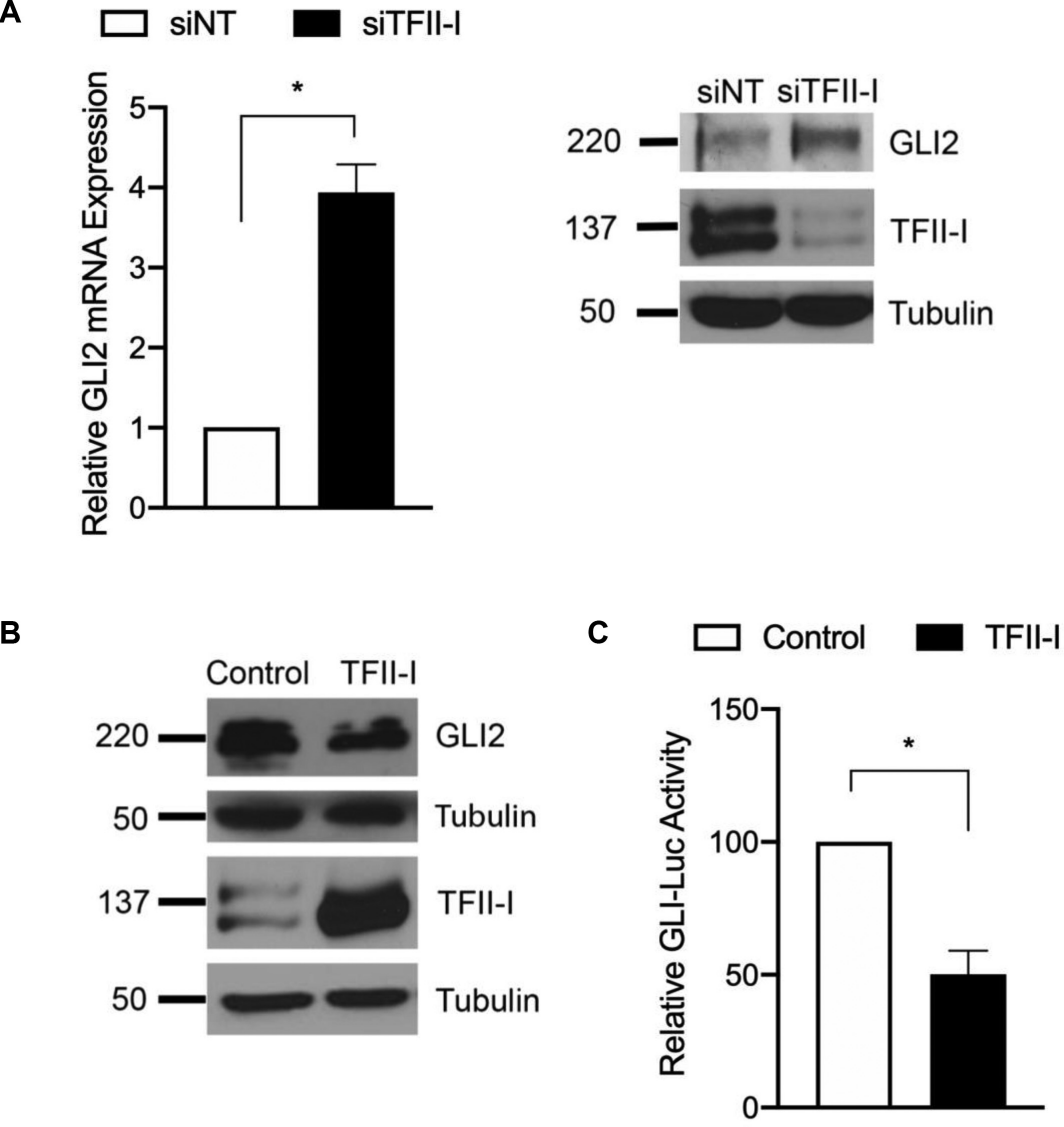
TFII-I represses GLI2 gene expression and subsequent function. (**A**) siRNA knockdown of TFII-I (siTFII-I) in HepG2 cells results in increased expression of GLI2 mRNA compared to non-targeting siRNA control (siNT). Samples were collected by 48 hours post-transfection and GLI2 RNA expression was determined by real time PCR (left), and GLI2 protein by western blot (right). (**B**) Overexpression of TFII-I cells results in decreased GLI2 protein expression. PANC1 cells were transfected for 24 h with a TFII-I encoding plasmid or vector only and then lysates were analyzed by western blot for GLI2 and TFII-I. Expression of TFII-I and tubulin is shown as well. (**C**) Co-transfection of TFII-I and the GLI-luc reporter in PANC1 cells demonstrates diminished activity of the GLI-responsive reporter. For this figure, *N* = 3 and the asterisk represents *P* ≤ 0.05.

To determine whether these repressive effects on the GLI2 promoter result in functional changes at the mRNA and protein levels, we knockdowned TFII-I using two independent targeting siRNA in HepG2 cells. These knockdowns resulted in increased GLI2 mRNA expression levels and a concomitant increase in protein (Figure 2A, and Supplemental Figure S1B). TFII-I knockdown expression controls are included in Figure 2A and Supplemental Figure S1B. Conversely, after overexpression of TFII-I in PANC1 cells we found decreased GLI2 protein expression (Figure 2B). These results suggest TFII-I acts as a repressor of GLI2 mRNA and protein expression. To further characterize this repressive mechanism, we evaluated if TFII-I -mediated repression of GLI2 led to decreased GLI2 transcriptional activity. We utilized an artificial GLI reporter system comprised of eight consecutive consensus GLI2-binding sites upstream of the luciferase gene (GLI-Luc). Luciferase expression is then dependent on this promoter, and the expression of luciferase can act as a proxy for measuring GLI2 activity. The results included in Figure 2C shows that TFII-I overexpression lowers GLI-Luc reporter activity. Taken together, these data from Figures 1 and 2 demonstrate a novel role for TFII-I as a repressor of GLI2 transcriptional activity through its binding to the INR region.

### TFII-I modulates RNA polymerase pausing without impacting chromatin modifications

**Figure 3.**
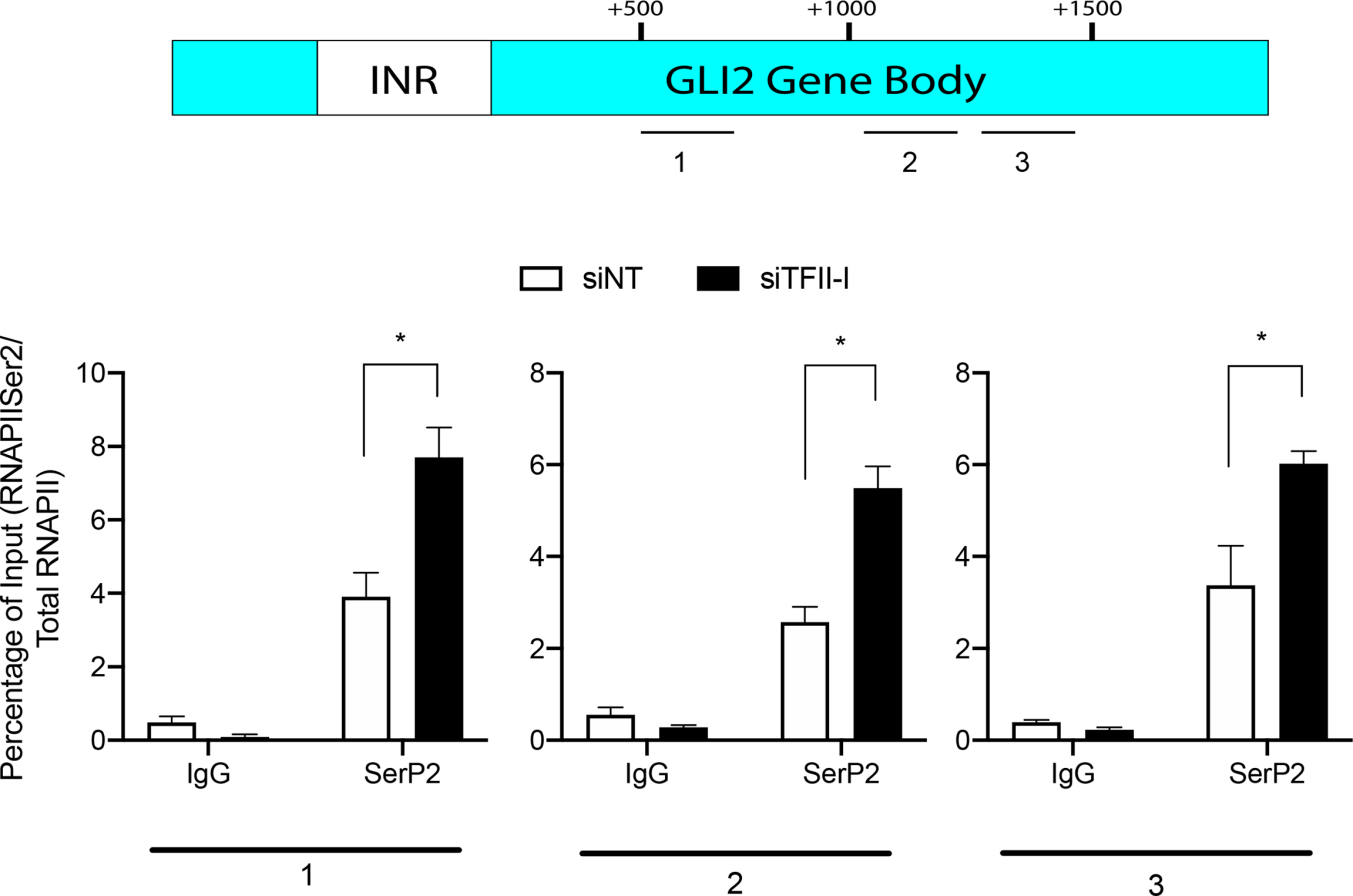
Inactivation of TFII-I increases RNA poll SerP2 levels in GLI2 gene body. RNAPII SerP2 enrichment in the promoter-proximal region of GLI2, ChIP assay was evaluated at three different areas in the gene body (1 through 3) (top). In all three regions, RNAPII SerP2 enrichment is increased upon TFII-I knockdown (bottom) in HepG2 cells. Data shown is a representative experiment of three performed showing the same trends. The asterisk represents *P* ≤ 0.05 in an evaluates of the representative data.

**Figure 4.**
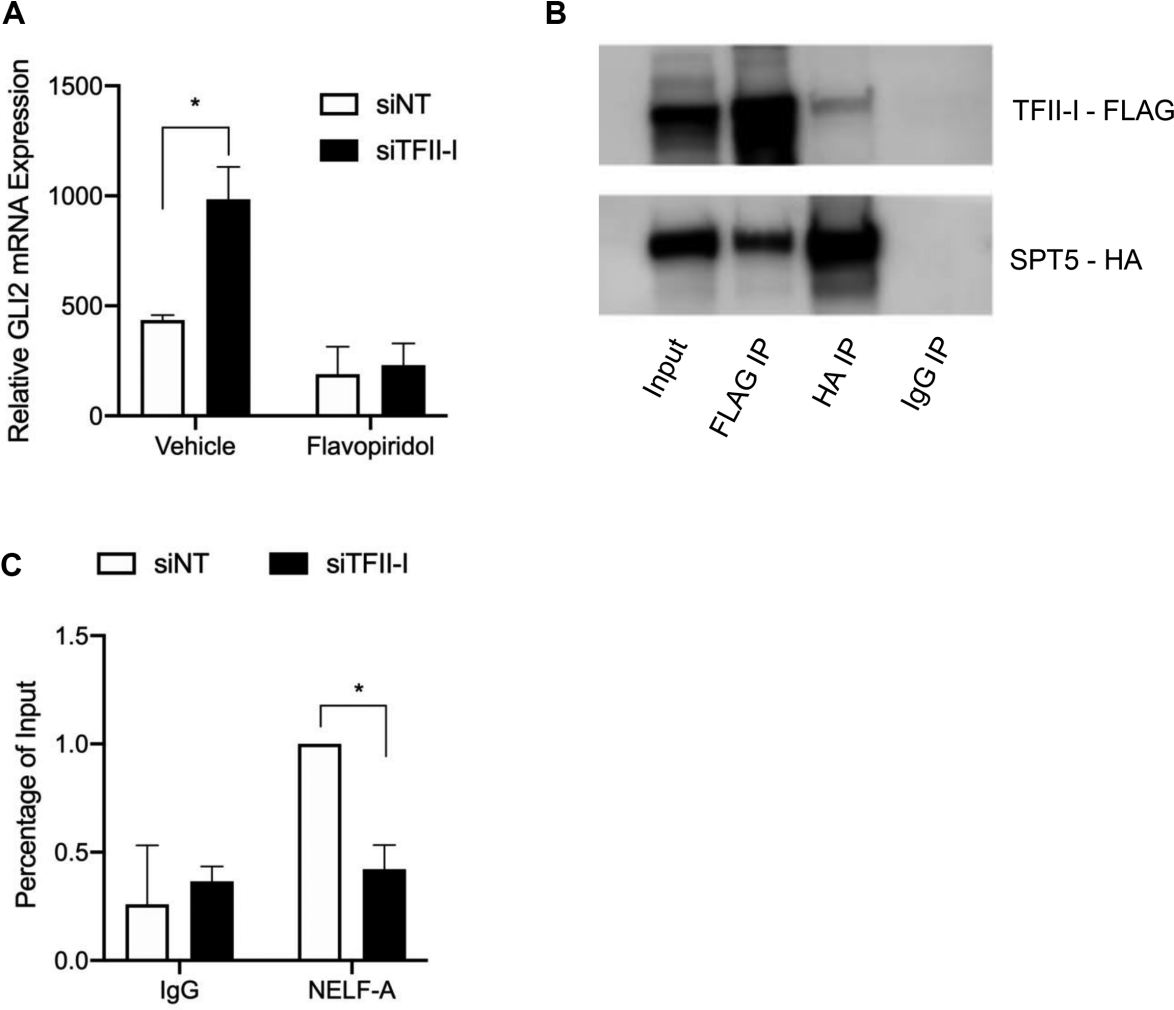
Knockdown of TFII-I releases RNAPII to transcribe GLI2. (**A**) Real time PCR showing GLI2 mRNA expression in PANC1 cells transfected with NT siRNA or siRNA targeting construct TFII-I and treated with vehicle or flavopiridol, an inhibitor of pause release. (**B**) Immunoprecipitation showing binding of endogenous TFII-I to SPT5. HepG2 cells transfected to express SPT5-HA and TFII-I-FLAG were immunoprecipitated with TFII-I antibody or nonspecific IgG control and then assayed by western blot. (**C**) ChIP assay was performed in HepG2 cells using an anti-NELF-A antibody. The data shows NELF-A I bind the INR of the GLI2 promoter, and that upon TFII-I knockdown endogenous NELF-A binding decreases at this region. These findings indicate TFII-I represses GLI2 gene transcription through RNAPII pausing. For this figure, *N* = 3 and the asterisk represents *P* ≤ 0.05.

Due to this repressive effect of TFII-I at the core promoter of GLI2 we sought to determine if TFII-I modulates this transcription factor expression by regulating RNAPII initiation or elongation. Knockdown of TFII-I did not affect RNAPII phosphorylated on serine 5 (RNAPII SerP5) or total RNAPII at the GLI2 promoter INR region (Supplemental Figure S3B, C). However, TFII-I depletion increased the phosphorylation of RNAPII on serine 2 (RNAPII SerP2), a mark of an actively transcribing RNAPII, in the GLI2 gene body (Figure 3, Supplemental Figure S3A). To investigate the importance of RNAPII pause release in mediating the effects of TFII-I, we utilized flavopiridol, a small molecule inhibitor of CDK9, which is the kinase required for phosphorylation of the serine 2 residue of RNAPII C-terminal domain (CTD) to promote entry into the active elongation phase. The effects of TFII-I knockdown on GLI2 expression was antagonized by flavopiridol, suggesting that the increase in GLI2 mRNA mediated by TFII-I depletion requires CDK9-mediated release of RNAPII from a paused state (Figure 4A). We also investigated physical interactions between components of the RNAPII pausing complex. We demonstrated that TFII-I physically interacts with SPT5 in HepG2 cells (Figure 4B). Intrestingly, we found that all four isoforms of TFII-I can co-immunoprecipitate with SPT5 (Supplemental Figure S2A). We next looked at the presence of the components of the RNAPII pausing complex on the GLI2 promoter after knocking down TFII-I. The increase in SerP2 resulting from TFII-I depletion (Figure 3) was accompanied by the decrease in the presence of NELF-A, a major component of the RNAPII pausing complex at the GLI2 gene INR (Figure 4C). Together, these findings suggest the repressive activity of TFII-I over GLI2 transcription occurs via physical interaction with the RNAPII pausing complex to promote polymerase pausing.

### TFII-I antagonizes TGFβ-mediated induction of GLI2

**Figure 5.**
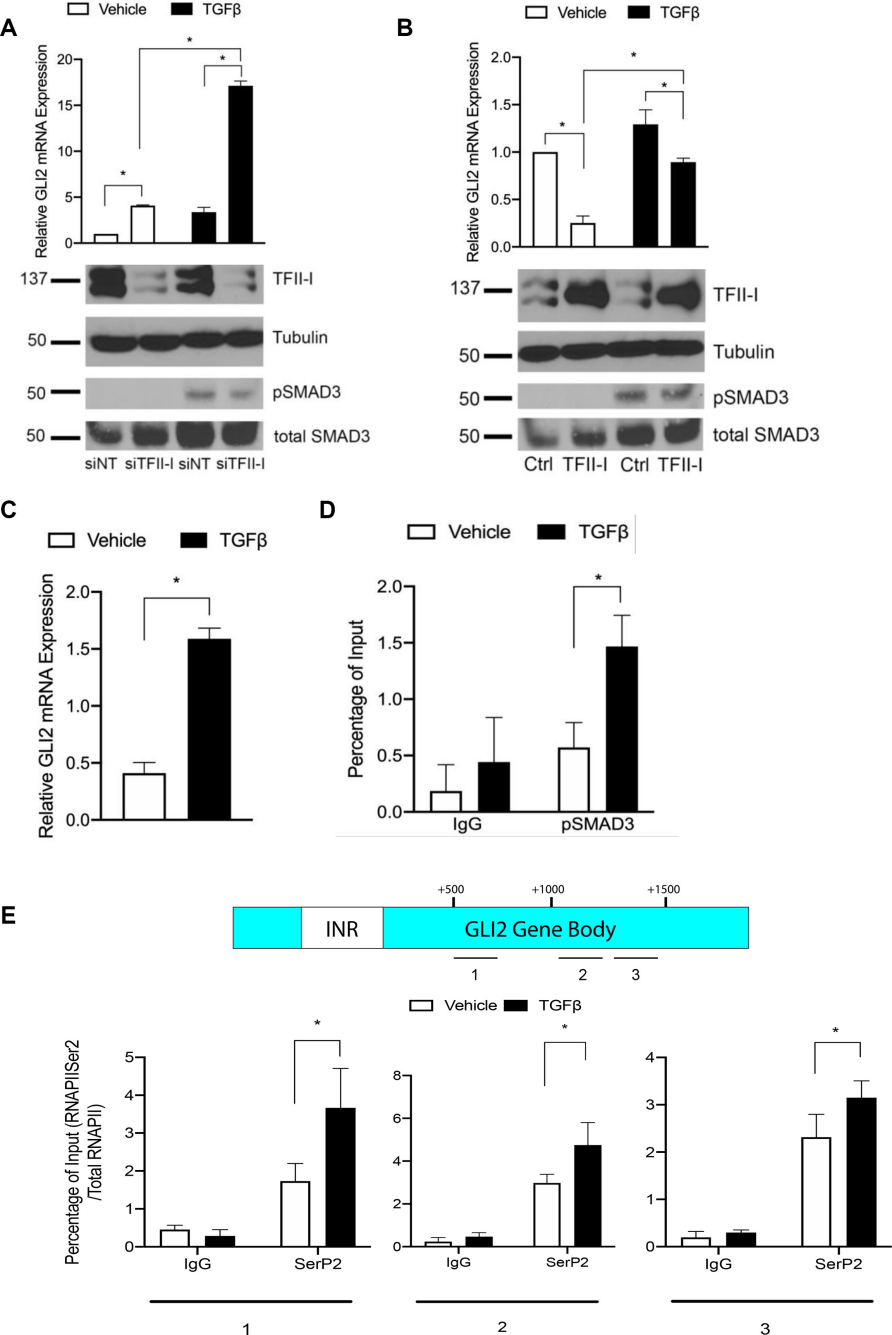
TFII-I antagonizes TGFβ-mediated induction of GLI2 transcription. (**A**) siRNA knockdown of TFII-I results in increased GLI2 mRNA that displays a synergistic effect when combined with TGFβ treatment in HepG2 cells. (**B**) Conversely, overexpression of TFII-I in PANC1 cells antagonizes the induction of GLI2 by TGFβ. Western blots show the levels of the TFII-I, phospho-SMAD3 (pSMAD3), total levels of SMAD3 (total SMAD3), and α-Tubulin. (**C**) Treatment with TGFβ induces GLI2 mRNA in HepG2 cells. (**D**) Treatment of HepG2 cells with TGFβ increases the binding of phosphorylated SMAD3 (pSMAD3) (left) while decreasing TFII-I binding to the GLI2 promoter INR (right). (**E**) ChIP assays reveal treatment with TGFβ increases RNAPII SerP2, particularly in the most promoter-proximal region, marking an actively elongating RNAPII and resulting in increased GLI2 transcript production in HepG2 cells. Data shown in E is a representative experiment of three performed showing the same trends. For A−D, *N* = 3 and the asterisk represents *P* ≤ 0.05.

Following the identification of TFII-I as a repressor of GLI2 transcription, we sought to determine if TFII-I can antagonize a known activator of GLI2 expression. Specifically, we investigated the possibility that TGFβ induction of GLI2 could be antagonized by TFII-I. HepG2 cells were transfected with siRNA against TFII-I or a non-targeting siRNA. Forty-eight hours later, these cells were treated with TGFβ for 3 h prior to harvest. GLI2 mRNA expression levels were then evaluated. While either stimulation of the cells with TGFβ or knockdown of TFII-I led to an increase in GLI2 expression, the combination of TGFβ pathway activation and loss of TFII-I resulted in a synergistic induction of GLI2 transcription (Figure 5A). However, when TFII-I was overexpressed in conjunction with TGFβ stimulation, GLI2 induction was mitigated (Figure 5B). Of note the overexpression or knockdown of TFII-I did not affect activity of TGFβ as indicated by the levels of phosphorylated SMAD3 (pSMAD3), the active form of this transcription factor and a key downstream effector of the TGFβ pathway (Figure 5A and B, lower panels). These results suggest that while TFII-I can repress GLI2 expression alone, it can also act as an antagonist of a known pathway of GLI2 induction. SMAD3 has been shown to bind at a SMAD binding element 30 bp upstream of the GLI2 transcription start site (26). To investigate the mechanism of TFII-I antagonism of TGFβ, we performed a ChIP assay in HepG2 cells following treatment with TGFβ. Treatment resulted in the predicted induction of GLI2 mRNA expression (Figure 5C) and increased binding of pSMAD3 to the GLI2 promoter (Figure 5D). To correlate these findings with that of a paused RNAPII associated with TFII-I, we performed ChIP analysis for RNAPII SerP2 downstream of the GLI2 INR in cells treated with TGFβ for 3 hours. We found that there is an increase in SerP2 in these regions upon GLI2 induction by TGFβ (Figure 5E). Taken together, these results demonstrate TFII-I can antagonize TGFβ induction of GLI2. Moreover, they show that TGFβ may induce GLI2 by reducing the binding of endogenous TFII-I to the GLI2 INR and releasing paused RNAPII.

**Figure 6.**
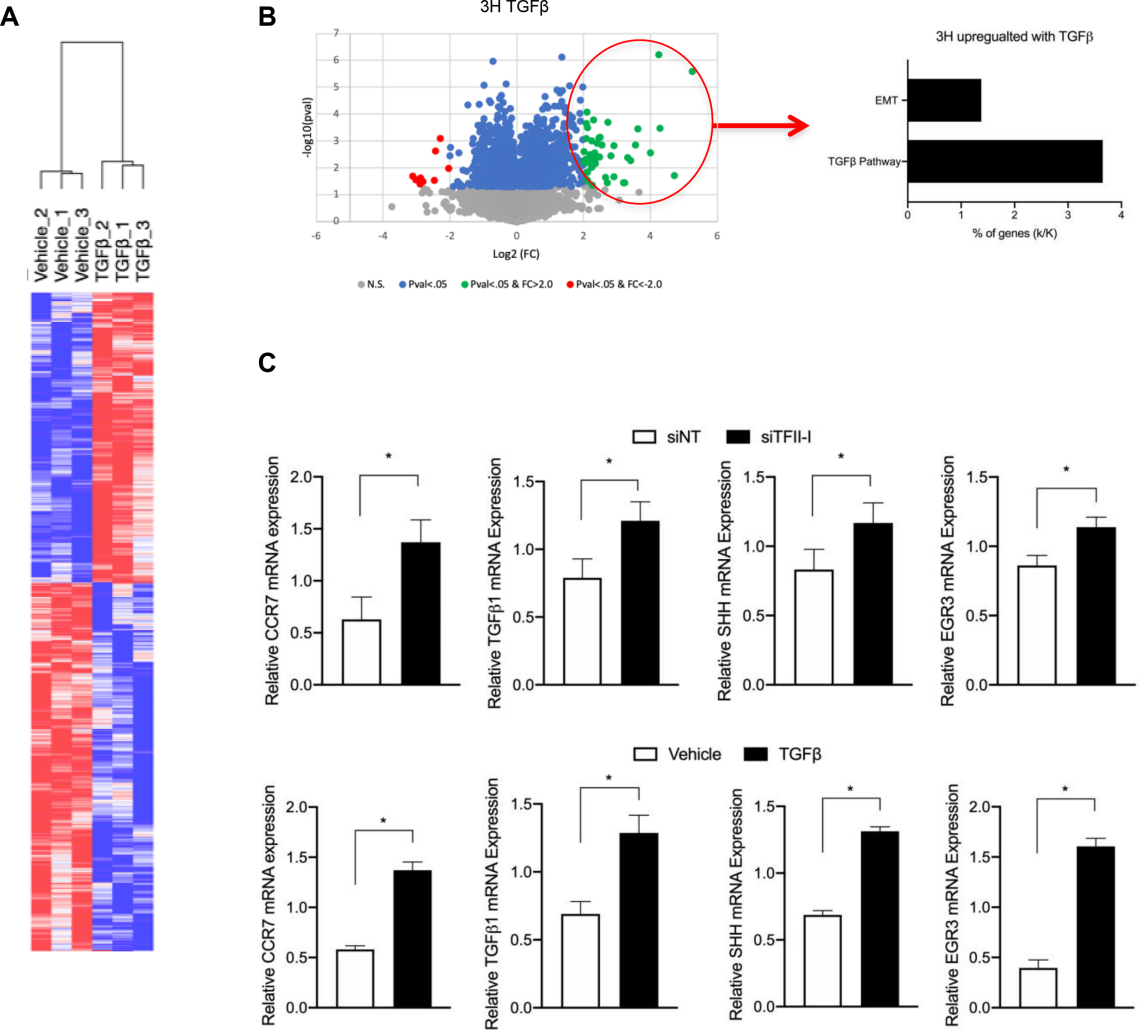
Interplay between TFII-I and TGFβ may be a regulatory mechanism for many genes in HepG2 cells. (**A**) Heatmap of RNA-seq data, displayed as log_2_ fold change. All genes with *P* < 0.05 between treatment and vehicle groups from RNA-seq analysis were included in the plot. Red represents increased values, blue represents decreased values compared to vehicle group. (**B**, left) RNA-seq data displayed in a volcano plot. *P* values are plotted as a function of fold change. Circled genes are those which display both a fold change greater than or equal to 2 between vehicle and TGFβ, and a *P* value of statistical significance (less than 0.05). (B, right) Pathway analysis of genes from A shown as percentage of genes from the input list (our data, represented as k) found in the pathway list (represented as K). (**C**) Induction of expression of a subset of genes that possess INRs and SMAD3 binding promoter sites after siRNA knockdown of TFII-I (top) or TGFβ treatment (bottom) was measured via qRT-PCR quantification of mRNA levels. For this figure, *N* = 3 and the asterisk represents *P* ≤ 0.05.

**Figure 7.**
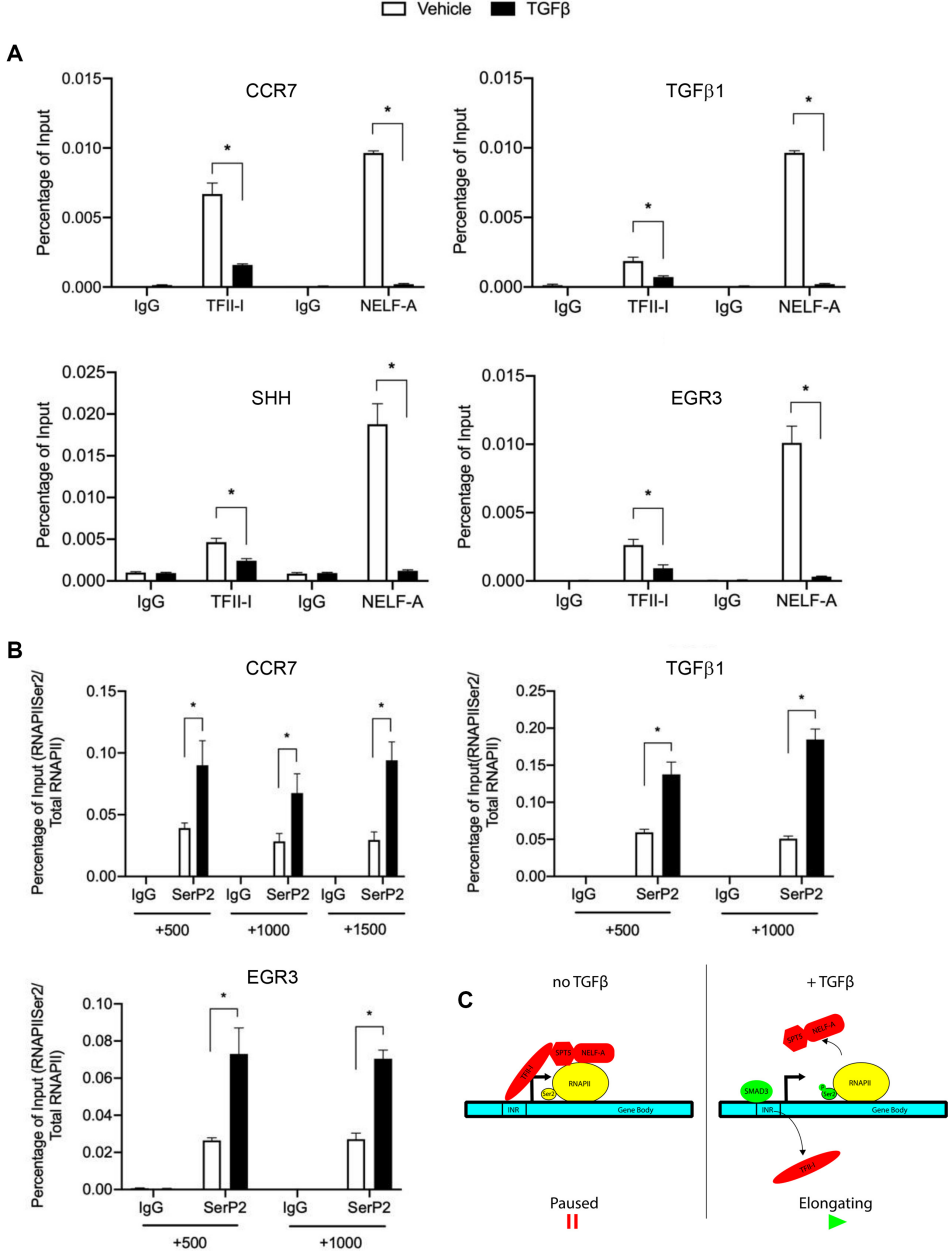
TGFβ induces an additional cohort of early response genes in HepG2 cells. (**A**) ChIP assays with TFII-I and a component of the pausing complex, NELF-A (**B**). Treatment with TGFβ increases RNAPII SerP2 throughout the gene body. (**C**) Graphic representation of our working model depicting the potential interaction between the TFII-I and TGFβ regulatory pathways. Data shown is a representative experiment of three performed showing the same trends. The asterisk represents *P* ≤ 0.05 in an evaluates of the representative data.

To determine whether this mechanism of gene regulation is present in other TGFβ-targets in HepG2 cells, we used RNA-seq data to assemble a list of TGFβ-induced genes for further investigation (Figure 6A). We narrowed our list down by comparing upregulated genes with known signature genes using GOterms (Figure 6B). From this list, we used Insect2.0 to find which in this list contained canonical SMAD3 binding motifs as well as an INR element, as we are interested in those genes that are regulated through the canonical TGFβ signaling pathway, and possibly bound by TFII-I, similar to GLI2. We determined that in addition to GLI2, TGFβ treatment and siRNA mediated TFII-I knockdown each induce the expression of CCR7, TGFβ1, SHH and EGR3 (Figure 6C). Finally, we demonstrated that upon treatment with TGFβ, members of the pausing complex (TFII-I and NELF-A) are dissociated from the promoter regions of CCR7, TGFβ1 and EGR3, (Figure 7A) and there is an increase in SerP2 in the gene bodies upon induction of expression (Figure 7B). Together, these results indicate our model of TFII-I mediated RNAPII pausing is applicable to a cohort of TGFβ-responsive genes (Figure 7C).

## DISCUSSION

GLI2 plays an important role in tumor development and maintenance, and it is found overexpressed in a variety of cancers. This oncogene regulates target genes that contribute to cell cycle progression, cell survival, and invasion and metastasis (3–11). Interestingly, genetic aberrations to explain this overexpression are uncommon. Thus, investigations into the regulatory mechanisms that increase GLI2 protein expression and transcriptional activation are necessary to better understand this phenomenon.

GLI2 is regulated through a variety of signaling pathways. In fact, GLI2 serves as a point of convergence for several pathways. Modulation of GLI2 protein activity and stability through the canonical Hedgehog pathway or noncanonical pathways, such as PI3K, has been investigated (28,29). However, regulatory mechanisms governing the transcriptional activation of GLI2 have been less studied. The TGFβ pathway is one of the few pathways studied shown to have direct transcriptional control over GLI2. Activation of the TGFβ pathway results in the binding of SMAD3 to a SMAD-binding element (SBE) in the promoter of GLI2 located 30 bp upstream of the transcription start site (26). Additionally, this pathway synergizes with the Wnt signaling pathway through the binding of TCF4/β-catenin at a TCF/LEF binding site near that of the SBE (26). While upregulation of these pathways plays a role in GLI2 activation, how these pathways activate GLI2 transcription is still unclear. The experiments described here add to our understanding of GLI2 regulation and provide insights into a regulatory mechanism that acts in a manner antagonistic to TGFβ.

Through knockdown experiments and ChIP assays, we have identified a novel repressive mechanism governing GLI2 transcriptional activity. This work finds that the ubiquitously expressed transcription factor TFII-I functions as a transcriptional repressor of GLI2 through maintenance of proximal promoter pausing. TFII-I has been shown to function as a transcriptional repressor via its ability to interact with histone modifiers that mark chromatin as inactive, such as HDAC1, HDAC3, and Suz12 of the PRC2 complex (18,21–24,30). However, our chromatin mapping and chemical inhibitor studies were unable to demonstrate the involvement of chromatin remodeling through histone modifications in TFII-I mediated regulation of GLI2 gene expression. Additional studies have indicated TFII-I may have a role in elongation, as it has tracked with elongating RNAPII throughout the gene body (31), however in the context of our investigations we observed dissociation of TFII-I in the promoter region and do not see binding in the gene body.

Promoter−proximal pausing of RNAPII has been identified as a regulatory mechanism of transcription for a large subset of genes (32–37). This mechanism of gene regulation has been highly investigated in more recent years following technological advances in ChIP-seq and global run-on (GRO) sequencing (32,33). The key finding in our work was the identification of RNAPII at the GLI2 transcription start site in cells that have both low and high basal expression levels of GLI2 mRNA. In HepG2 cells where GLI2 expression is minimal but rapidly induced by TGFβ, we saw an increase in RNAPII SerP2 in the gene body upon knockdown of TFII-I. This is consistent with a release of RNAPII pausing to resume elongation of the gene. What has not been definitively determined from this mechanism is the state in which RNAPII was paused. One possibility is that the recruited RNAPII may have been poised for transcription but never proceeded to the elongation step. Alternatively, the RNAPII may have initiated elongation but was stalled early in this process. Given the rapid induction of GLI2 transcription secondary to treatment of cells with TGFβ ligand, it may be possible that the latter scenario is occurring. However, further investigations are needed to show this. Regardless, similar changes were seen in the location and phosphorylation status of RNAPII upon TGFβ activation as were identified with TFII-I knockdown. Previous work has indicated a role of the TGFβ signaling pathway in phosphorylating and/or regulating the transcriptional activity of TFII-I (38,39). Our findings suggest that pSMAD3 binding relieves TFII-I binding and thereby allows release of RNAPII (Figure 7C). While it is evident that relief from TFII-I binding to the INR of GLI2 results in the active transcription of GLI2, the role of specific TFII-I isoforms has not been addressed. Lastly, we have not investigated how TFII-I may be regulated in these cells. The most studied pathway of TFII-I activation is that of interaction with Bruton's tyrosine kinase, but this is specific to B-lymphocytes (40,41). TFII-I may also be activated and regulate its target genes via PI3K/AKT or TGFβ signaling (42–45). Alterations in TFII-I regulation/phosphorylation could contribute to more or less binding of TFII-I to promoters such as at the GLI2 locus and thereby change their transcriptional status through modulating RNAPII pausing.

In conclusion, the study presented here outlines a novel mechanism of TFII-I mediated transcriptional repression of GLI2 through binding the INR. To our knowledge, this is the first reported mechanism of transcriptional repression of GLI2. The significance of these findings is enhanced by the fact that TFII-I is able to antagonize a known inducer of GLI2 transcription, TGFβ. Continued studies into how TFII-I activity may be modulated in cancer cells may lead to new avenues through which GLI2 expression can be inhibited. Given its relevance in tumorigenesis, inhibiting GLI2 may be a reasonable target for therapeutic interventions, and our understanding of how TFII-I represses GLI2 expression furthers our insight into the mechanisms of GLI2 transcriptional regulation. Furthermore, this mechanism of gene transcriptional regulation was shown to be applicable to a subset of tumor supportive genes and identifies TFII-I as an important factor in the regulation of RNAPII pausing.

## DATA AVAILABILITY

GEO: GSE139021.

## Supplementary Material

gkaa476_Supplemental_File

## Notes

Present address: Angela L. McCleary-Wheeler, Veterinary Health Center, University of Missouri, Columbia, MO 65211, USA.
