# Supplementary material for: TFII-I-mediated polymerase pausing antagonizes GLI2 induction by TGFβ
Source: Nucleic Acids Res. 2020 Jun 16;48(13):7169–81. doi: 10.1093/nar/gkaa476 (PMC7367210; doi:10.1093/nar/gkaa476)
Supplement: gkaa476_Supplemental_File [file gkaa476_supplemental_file.pdf]

Supplementary Figure 1

A

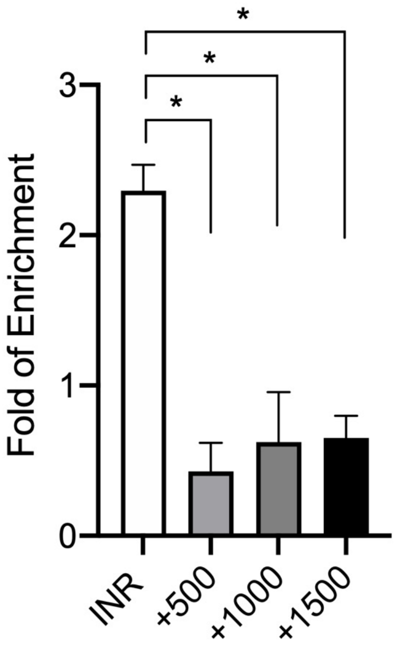

B

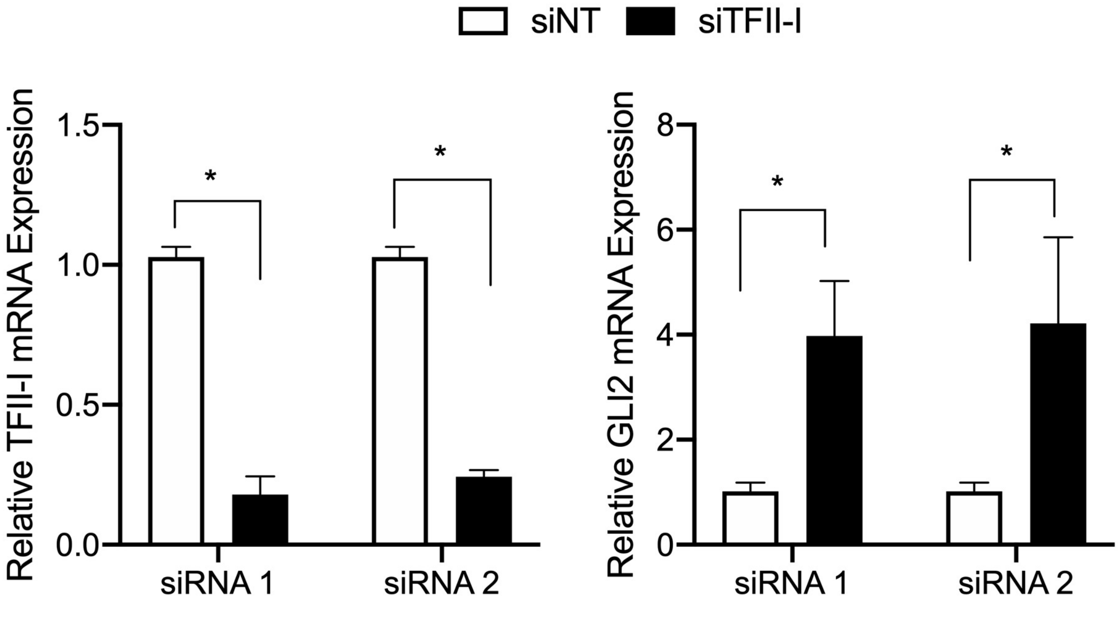

Supplementary Figure 2

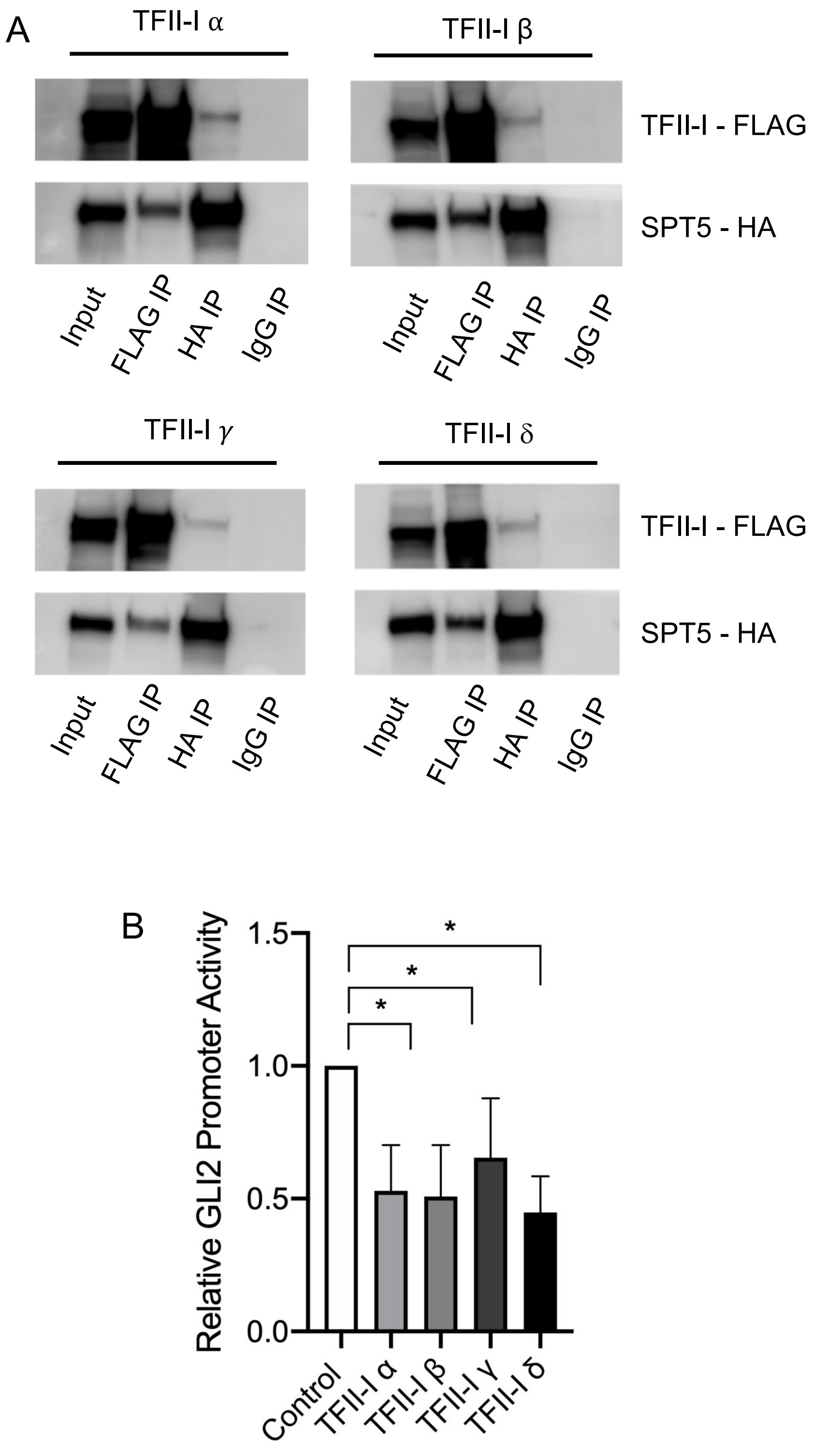

Supplementary Figure 3

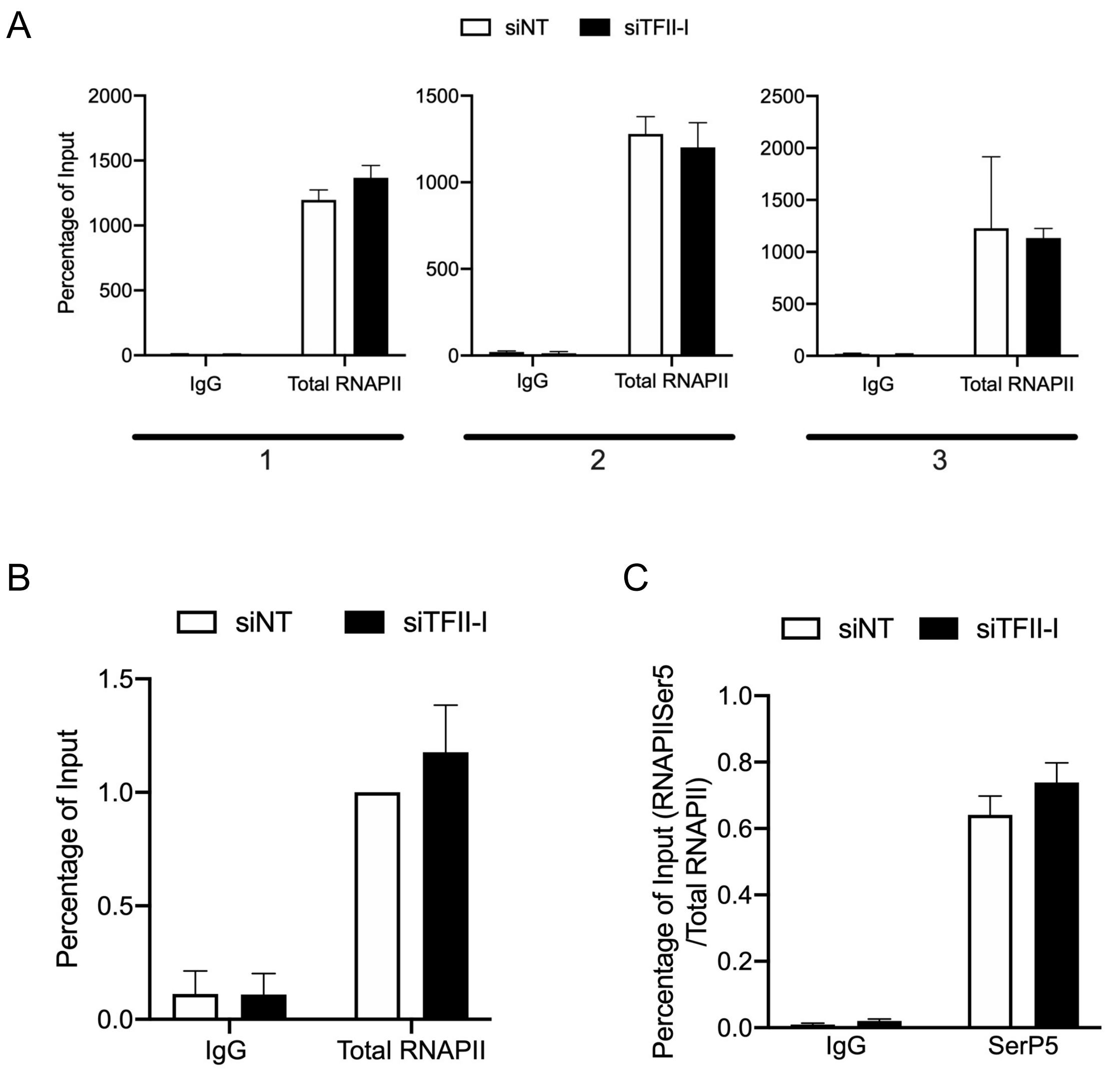

Supplemental Figures Legends

Supplemental Figure 1. TFII-I binds to the GLI2 promoter's INR and act as a repressor of GLI2 expression. (A) TFII-I binding specificity for the INR was evaluated using chromatin immunoprecipitation (ChIP) assays. The fold of enrichment of TFII-I binding to the INR is approximately 4-fold higher than the +500, +1000, and +1500 regions upstream of the TSS. Data shown the average of N=3 and asterisk represents $p \leq 0.05$. (B) In HepG2 cells, a second siRNA knockdown of TFII-I (siRNA 1) using a different targeting sequence was utilized to validate the effects of TFII-I on GLI2 mRNA expression. Quantified by RT-qPCR, TFII-I levels decreased and GLI2 mRNA expression increased to a similar extent between two different siRNAs used. (siRNA 1 for validation, as siRNA 2 is used throughout the paper). For this figure, N=3 and asterisk represents $p \leq 0.05$.

Supplemental Figure 2. TFII-I isoforms α, β and γ resemble the δ isoform repressor function. (A) The TFII-I isoforms α, β and γ isoforms were FLAG tagged and overexpressed in HepG2 cells together with SPT5-HA. Immunoprecipitation assays using FLAG, HA, of IgG control were performed and evaluated by western blotting. The data show that each isoform can co-immunoprecipitated with SPT5. (B) The indicated isoforms were overexpressed with GLI2-Full length luciferase reporter in PANC1 cells. Luciferase activity, as measure of the promoter activity, shows that each TFII-I isoform can represses GLI2 promoter to a similar extent. For this figure, N=3 and the asterisk represents $p \leq 0.05$.

Supplemental Figure 3. Knockdown of TFII-I does not affect total RNAPII binding. (A-B) siRNA mediated targeting of TFII-I does not change total RNAPII binging in the (A) GLI2 gene body at three different areas in the gene body (1 through 3) or (B) GLI2 core promoter examined by ChIP assays. (C) ChIP assay shows that the levels phosphorylation of serine 5 in the core promoter region of GLI2 is not affected by TFII-I knockdown. The data in this figure is a representative of one of three biological replicates.
